# Single Low-Volume Center Experience with Frozen Elephant Trunk in Acute Type A Aortic Dissections

**DOI:** 10.1055/s-0039-1677809

**Published:** 2019-04-24

**Authors:** Luis F. López Almodóvar, Pedro Lima Cañadas, Andrés Enríquez Puga, Irene Narváez Mayorga, José A. Buendía Miñano, Marcelino Sánchez Casado, Alfonso Cañas Cañas

**Affiliations:** 1 Department of Cardiac Surgery, Virgen de la Salud Hospital, Toledo, Spain; 2 Department of Radiology, Virgen de la Salud Hospital, Toledo, Spain; 3 Department of Cardiology, Virgen de la Salud Hospital, Toledo, Spain; 4 Department of Intensive Care Unit, Virgen de la Salud Hospital, Toledo, Spain

**Keywords:** aortic dissection, aorta, frozen elephant trunk

## Abstract

**Background** Acute Type A aortic dissection (AAAD) is a surgical emergency. In patients with arch and descending aorta involvement (DeBakey Type I), a total aortic arch replacement with frozen elephant trunk (FET) could favor false lumen thrombosis and improve long-term results. The authors hereby present their experience with this technique in a single low-volume center, to assess whether the technique is feasible to treat such disease.

**Methods** From January 2011 to December 2016, 43 patients with AAAD were operated on in the authors' institution, which carries out 300 to 350 annual procedures. Among these, 12 patients with an intimal tear in the aortic arch and/or proximal descending aorta received a FET procedure (10 males, age 57 years). Concomitant procedures were aortic valve replacement (42%), Bentall (25%), and aortic valve repair (17%).

**Results** Cardiopulmonary bypass, cardiac arrest, and circulatory arrest times were 235 ± 43, 171 ± 33, and 75 ± 20 minutes, respectively. The operative mortality was 16.7% ( *n* = 2). Stroke and re-thoracotomy for bleeding occurred in 8% ( *n* = 1) and 8% ( *n* = 1), respectively. There was no spinal cord injury. Follow-up was 36.1 months. During follow-up, no patients died or required a reoperation on the downstream aorta.

**Conclusion** Although all patients were operated on in a low-volume center, the results with FET in AAAD are acceptable. Even though this technique demands high technical skills, it is a promising approach in patients with acute aortic dissection.

## Introduction

Acute Type A aortic dissection (AAAD) is an absolute emergency with a high mortality rate despite surgical therapy. Although the outcome of surgical repair has improved through last advances, operative mortality is still high and many surgeons advocate only an ascending aortic replacement with or without replacement of the proximal arch. However, in DeBakey Type I aortic dissection, which involves the aortic arch and descending aorta, there is still no consensus regarding the most appropriate surgical approach for the treatment. 1

The arch replacement combined with antegrade stent grafting into the descending aorta (called *frozen elephant trunk* [FET] technique) is being used for the treatment of DeBakey Type I aortic dissection in some experienced centers to favor false lumen thrombosis around the stent graft, 2 3 because a patent false lumen was identified as a risk factor for aortic dilatation and late operation. 4

Ideally, these operations should be done in experienced centers. 5 The purpose of this study was to assess the initial results of FET technique for the treatment of AAAD in a single low-volume center (mean incidence of AAAD 7–9 patients annually).

## Materials and Methods

Between January 2011 and December 2016, 43 consecutive patients underwent surgical treatment for AAAD in our Institution. Among these, 12 (28%) patients with an intimal tear in the aortic arch and/or proximal descending aorta underwent FET procedure and were included in our analysis. Almost all cases (11/12) were performed by a single thoracic aortic surgeon, who did a mini-fellowship training in FET surgery.

Data were prospectively collected in a database, and the retrospective review was approved by the local ethics committee. Individual patient consent was waived.

Preoperative demographic patient characteristics are shown in Table 1 . Coma was defined as complete mental unresponsiveness, with no evidence of psychological responses to stimulation, due to arch vessel involvement by the dissection.

**Table 1:** Preoperative data

Variables	Value (Percentage)
Total patients, *n*	12
Sex male, *n* (%)	10 (83)
Age (y)	57.11
Hypertension, *n* (%)	10 (83)
Coma, *n* (%)	1 (8)
Reoperation, *n* (%)	2 (17)
Malperfusion, *n* (%)	
Penn a, *n* (%)	6 (50)
Penn b, *n* (%)	1 (8)
Penn c, *n* (%)	2 (17)
Penn b + c, *n* (%)	3 (25)
Hemopericardium, *n* (%)	4 (33)
Aortic regurgitation III-IV, *n* (%)	7 (58)
EuroSCORE logistic (%)	22.41

Note: Continuous data summarized as mean; categorical and binary data summarized as frequency (percentage).

Patients were followed every 6 months the first 2 years, and then annual. Clinical follow-up ended in December 2016 and was 100% complete.

Our principal strategy for the treatment of AAAD involves the resection of the primary entry, usually in the ascending aorta. Nevertheless, in patients with entry in the distal aortic arch/proximal descending aorta, the FET technique was performed. The detailed operative procedure has been described previously. 6 7 8 9 Briefly, when a target nasopharyngeal temperature of 25°C was achieved, the ascending aorta was clamped and cardioplegia was administered. Concomitant aortic root procedures were performed, if necessary. At this time, the pump flow was reduced to 0.8 to 1 L, the innominate artery was clamped, the aortic clamp was removed, and under unilateral right brain perfusion, the aorta was opened. Bilateral antegrade cerebral perfusion was established with a flow of 10 mL/kg/min, after endoluminal cannulation of the left carotid artery and left subclavian artery. After complete resection of the aortic arch, FET was performed with an E-vita Hybrid Open Plus stent graft (Jotec). The sizing of the stent-graft was done according to the dimension of the true thoracic aortic lumen at the level of the pulmonary bifurcation without oversizing. The length of the stent graft was 130 mm. The false lumen was obliterated using four pledgeted 3–0 polypropylene U-stitches with a Teflon felt on the outside of the aorta. Glue, sealants, or adhesives were not used. After island reimplantation of the epiaortic vessels, the clamp from the innominate artery was removed, de-airing was carefully performed, and antegrade cardiopulmonary bypass was reestablished by clamping the Dacron graft at its proximal end. Aortic root procedure was completed. Finally, cardiopulmonary bypass was weaned as usual. In acute cases, we do not use cerebrospinal drain.

**Fig. 1:**
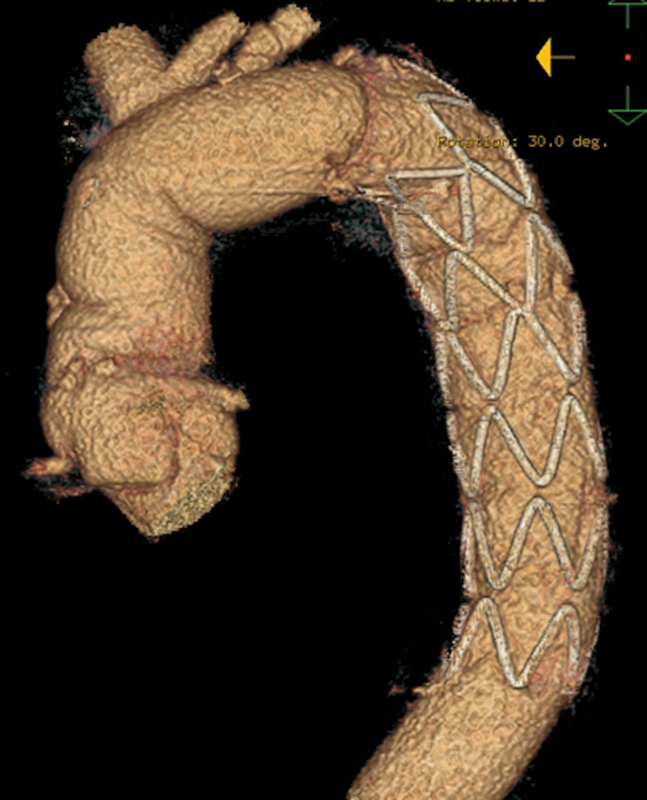
Frozen elephant trunk (FET) procedure with island technique.

Follow-up computed tomography (CT) examinations were performed postoperatively, every 6 months for 2 years, and yearly thereafter ( Fig. 1 ). A follow-up CT image was available for 100% of patients. The average total duration of follow-up was 36.1 ± 28.7 months.

### Statistical Analysis

All data were retrospectively analyzed. SPSS 23 (SPSS) was used to performed data analysis. Continuous data were expressed as mean ± standard deviation (SD) if there was normal distribution or median ± interquartile range if not. Categorical data were expressed as numbers and percentages. The Kaplan-Meier survival estimate was used to analyze survival.

## Results

The detailed intraoperative data and perioperative results are described in Tables 2 and 3 .

**Table 2:** Operative data

Variables	Total ( *n* = 12)
CBP time (min)	235 ± 43
Cardiac arrest time (min)	171 ± 33
Circulatory arrest time (min)	75 ± 20
SACP time (min)	96 ± 23
Concomitant procedures	
Aortic valve resuspension	2 (17%)
Aortic valve replacement	5 (42%)
Bentall	3 (25%)
Evita open stent graft 130 mm	
24 mm	1 (8%)
28 mm	6 (50%)
30 mm	4 (34%)
33 mm	1 (8%)
Head-vessel reimplantation	
Bloc	11 (92%)
Separately	1 (8%)
Left subclavian artery rerouting	6 (50%)

Abbreviations: CPB, cardiopulmonary bypass; SACP, selective antegrade cerebral perfusion.

Note: Continuous data summarized as mean ± standard deviation (SD); categorical and binary data summarized as frequency (percentage).

**Table 3:** Perioperative results

Variables	Total ( *n* = 12)
30-day mortality	2 (16.7%)
In-hospital mortality	2 (16.7%)
Re-thoracotomy (bleeding)	1 (8.3%)
Paraplegia	0 (0%)
Prolonged mechanical ventilation > 72 h	2 (16.7%)
Temporary dialysis	1 (8.3%)
Stroke	1 (8.3%)
Recurrence nerve palsy	0 (0%)

Note: Binary data summarized as frequency (percentage).

Stroke was defined as the persistent loss of neurologic function caused by an ischemic event, with confirmation by CT imaging after surgery.

**Fig. 2:**
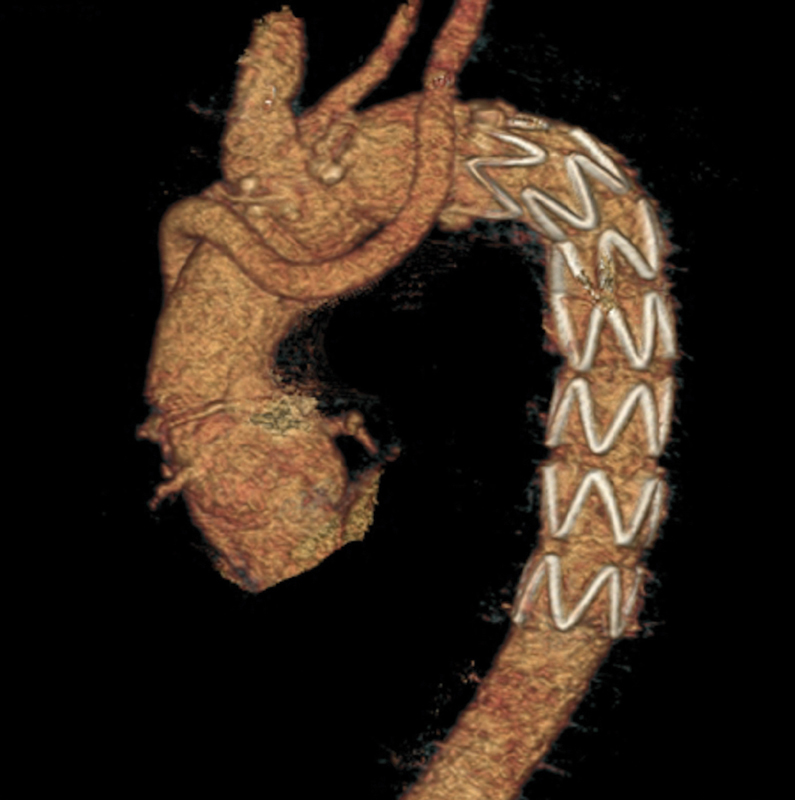
FET procedure with reroute of the left subclavian artery.

In-hospital mortality rate was 17% (2/12). Both were intraoperative deaths, because of bleeding around the origin of the left subclavian artery (aortic wall), despite all efforts. After this, we decided to reroute the left subclavian artery separately by end-to-end anastomosis between the origin of the left subclavian artery and an 8-mm vascular graft, and to remove the distal anastomosis to zone 2 ( Fig. 2 ). 10 There were no other in-hospital deaths after this improvement.

**Fig. 3:**
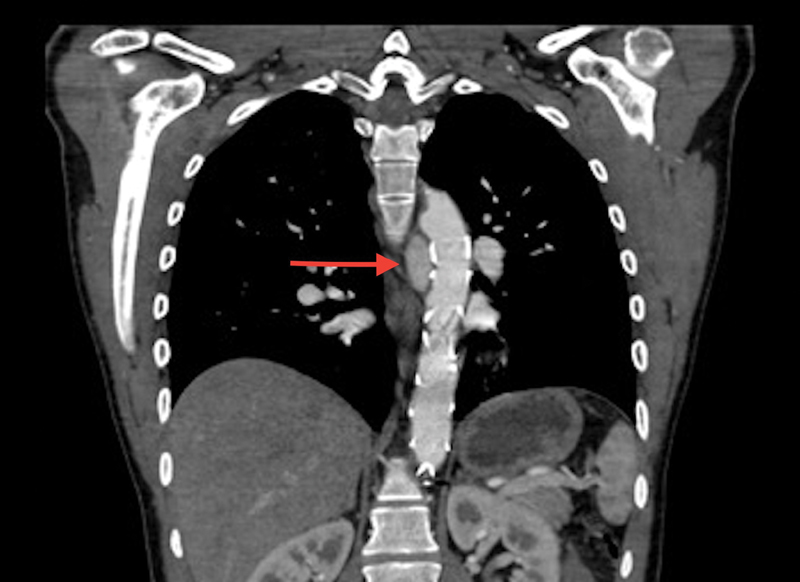
Thoracic endoleak type II (red arrow).

During follow-up, no patients died or required a reoperation on the downstream aorta. One patient had a pseudoaneurysm at the level of the proximal anastomosis, just above the origin of the right coronary artery, resolved spontaneously. Another patient had an endoleak type II at the level of the thoracic stent graft; after conservative management, the endoleak disappeared completely 1 year later ( Fig. 3 ).

**Fig. 4:**
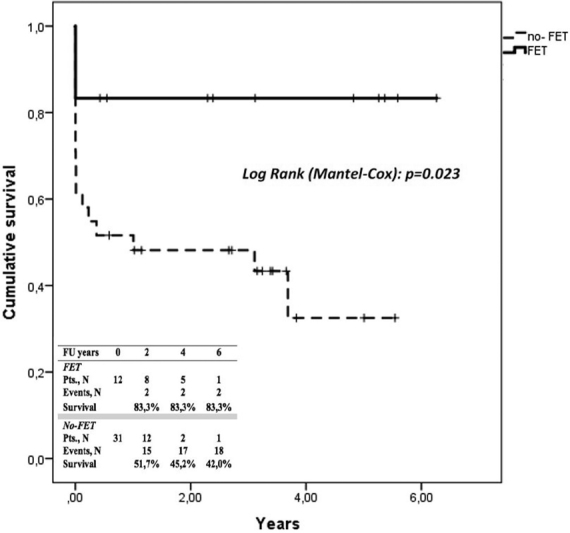
Kaplan-Meier curves showing overall cumulative survival in acute Type A aortic dissection (AAAD) in our Institution, with and without frozen elephant trunk (FET) technique.

Compared with patients who underwent conventional techniques in our institution during the same observation period, we demonstrate a reduction in operative mortality in FET patients, improving overall 6-year actuarial survival rates ( Fig. 4 ).

## Discussion

The operation for AAAD, which is a surgical emergency, should be kept as simple as possible, as the main goal is to save the life. In most patients, this can be achieved by the replacement of the ascending aorta and closure of the primary entry tear, because it is known that the primary entry tear usually originates in the ascending aorta.

However, in DeBakey Type I survivors, there is a persistent distal aortic dissection, with up to 89% patent false lumen reported that enlarge over time. A significant proportion of these patients requires another operation or dies from this reason; therefore, an aggressive treatment might be required for improving the long-term outcomes of cases of AAAD. 2 11

To prevent this, some groups have proposed a total aortic arch replacement with FET implantation to stabilize the dissecting membrane in the proximal descending aorta and seal the false lumen to prevent its dilatation. 12 13 14 However, in patients with DeBakey Type I acute dissection, performing the FET technique is still controversial.

Treatment with FET technique in patients with AAAD has a significant positive remodeling effect not only on the stented segment but also on the downstream aorta, although this effect decreases with the distance from the stent graft. 15 FET can achieve closure of the primary entry even if the entry occurs in the proximal descending aorta. Closure of the primary tear excludes the antegrade blood flow in the false lumen and achieves expansion of the true lumen, avoiding malperfusion of abdominal visceral branches. Furthermore, the thoracic stent graft might expand the true lumen because of its radial self-expansion force. This fact is very important in acute perioperative period, because the appearance of a malperfusion syndrome has a great impact on the prognosis of the patient. In addition, in the follow-up, the false lumen thrombosis is related with less incidence of aneurysm. 16

Indications for FET remains controversial. Although the main indication is still a reentry or tear in the distal aortic arch or proximal descending aorta, some authors have proposed that primary indication for the FET technique should be patients younger than 70 years. 17 Other authors, in line with the current recommendation of The European Association for Cardio-Thoracic Surgery expert group, 18 have pointed out that patients with AAAD DeBakey Type I who meet at least one of the following criteria could be treated with FET technique: lower body malperfusion, reentry or tear in the distal aortic arch/proximal descending aorta, or patients younger than 70 years. 19

On the other hand, we strongly feel that, owing to low volume of general cardiac surgery in our institution, the hospital mortality for AAAD was excessive, at around 40%. In response to perceived poor outcomes from AAAD repair, in 2011, to reduce operative mortality and associated morbidity, several perioperative factors changed. This evolution was initiated by a senior surgeon who began, among other things, with the implementation of the FET technique. Ideally, these operations should be done in experienced centers that can do this type of surgery in selected patients. Some authors have advocated that the surgeon should have performed at least 20 elective FET implantations before he/she starts with such an operation in an acute dissection patient. 5 However, this scenario is not always possible. 20 Although interpreting results from a single low-volume center is very different to results from multiple low-volume centers, and we would probably require an analysis of results from multiple low-volume centers to improve the reliability of the results, our results clearly demonstrate a reduction in operative mortality in our institution in patients with AAAD after implementation of the FET procedure, among other reasons due to the improvement of the surgical management. This improvement in survival is likewise demonstrated in the 6-year actuarial survival rates. In addition, there is a reduction in operative mortality in AAAD patients in which classic technique is performed during the same period. Deaths occurred in the first cases, by technical aspects related to the learning curve that have been improved.

## Limitations

The main limitation of this study is the retrospective nature to the data analysis, apart from our small subset of patients. Prospective randomized trials have never been performed in AAAD. However, we think it is enough for another centers that want to start an aortic program that includes FET technique for the treatment of patients with AAAD. The development of standardized surgical techniques and the increased number of cases per surgeon are among the main contributing factors to the benefits demonstrated, including improved midterm survival.

## Conclusion

Although FET technique in patients with DeBakey Type I acute aortic dissection demands high technical skills, the implantation of an FET in these patients is helpful because it expands the true lumen avoiding malperfusion syndromes and prevents future events on the distal aorta. Even though this technique demands high technical skills, this technique might be carried out in low-volume centers as well, with acceptable results, and it is a promising approach in patients with acute aortic dissection.

## References

[1] Bonser R S Ranasinghe A M Loubani M Evidence, lack of evidence, controversy, and debate in the provision and performance of the surgery of acute type A aortic dissection J Am Coll Cardiol 2011 58 24 2455 2474 22133845 10.1016/j.jacc.2011.06.067

[2] Smith H N Boodhwani M Ouzounian M Classification and outcomes of extended arch repair for acute type A aortic dissection: a systematic review and meta-analysis Interact Cardiovasc Thorac Surg 2017 24 03 450 459 28040765 10.1093/icvts/ivw355

[3] Tian D H Wan B Di Eusanio M Black D Yan T D A systematic review and meta-analysis on the safety and efficacy of the frozen elephant trunk technique in aortic arch surgery Ann Cardiothorac Surg 2013 2 05 581 591 24109565 10.3978/j.issn.2225-319X.2013.09.07 PMC3791206

[4] Li D Ye L He Y False lumen status in patients with acute aortic dissection: a systematic review and meta-analysis J Am Heart Assoc 2016 5 05 e003172 27166218 10.1161/JAHA.115.003172 PMC4889188

[5] Shrestha M Fleissner F Ius F Total aortic arch replacement with frozen elephant trunk in acute type A aortic dissections: are we pushing the limits too far? Eur J Cardiothorac Surg 2015 47 02 361 366, discussion 366 24829403 10.1093/ejcts/ezu185

[6] Pacini D Tsagakis K Jakob H The frozen elephant trunk for the treatment of chronic dissection of the thoracic aorta: a multicenter experience Ann Thorac Surg 2011 92 05 1663 1670, discussion 1670 22051263 10.1016/j.athoracsur.2011.06.027

[7] Tsagakis K Pacini D Di Bartolomeo R Multicenter early experience with extended aortic repair in acute aortic dissection: is simultaneous descending stent grafting justified? J Thorac Cardiovasc Surg 2010 140(6, Suppl):S116–S120, discussion S142–S146 21092776 10.1016/j.jtcvs.2010.07.066

[8] Czerny M Rylski B Kari F A Technical details making aortic arch replacement a safe procedure using the Thoraflex™ Hybrid prosthesis Eur J Cardiothorac Surg 2017 51 01 i15 i19 28108564 10.1093/ejcts/ezw303

[9] Mestres C A Tsagakis K Pacini D One-stage repair in complex multisegmental thoracic aneurysmal disease: results of a multicentre study Eur J Cardiothorac Surg 2013 44 05 e325 e331 23918768 10.1093/ejcts/ezt374

[10] Tsagakis K Dohle D Benedik J Lieder H Jakob H Overall Essen's experience with the E-vita open hybrid stent graft system and evolution of the surgical technique Ann Cardiothorac Surg 2013 2 05 612 620 24109569 10.3978/j.issn.2225-319X.2013.09.17 PMC3791188

[11] Kazui T Washiyama N Muhammad B A Extended total arch replacement for acute type a aortic dissection: experience with seventy patients J Thorac Cardiovasc Surg 2000 119 03 558 565 10694617 10.1016/s0022-5223(00)70136-x

[12] Leontyev S Tsagakis K Pacini D Impact of clinical factors and surgical techniques on early outcome of patients treated with frozen elephant trunk technique by using EVITA open stent-graft: results of a multicentre study Eur J Cardiothorac Surg 2016 49 02 660 666 25890937 10.1093/ejcts/ezv150

[13] Leontyev S Borger M A Etz C D Experience with the conventional and frozen elephant trunk techniques: a single-centre study Eur J Cardiothorac Surg 2013 44 06 1076 1082, discussion 1083 23677901 10.1093/ejcts/ezt252

[14] Di Bartolomeo R Murana G Di Marco L Frozen versus conventional elephant trunk technique: application in clinical practice Eur J Cardiothorac Surg 2017 51 01 i20 i28 28108565 10.1093/ejcts/ezw335

[15] Dohle D S Tsagakis K Janosi R A Aortic remodelling in aortic dissection after frozen elephant trunk Eur J Cardiothorac Surg 2016 49 01 111 117 25715431 10.1093/ejcts/ezv045

[16] Jakob H Dohle D Benedik J Long-term experience with the E-vita Open hybrid graft in complex thoracic aortic disease Eur J Cardiothorac Surg 2017 51 02 329 338 28082472 10.1093/ejcts/ezw340

[17] Katayama A Uchida N Katayama K Arakawa M Sueda T The frozen elephant trunk technique for acute type A aortic dissection: results from 15 years of experience Eur J Cardiothorac Surg 2015 47 02 355 360, discussion 360 24801338 10.1093/ejcts/ezu173

[18] Shrestha M Bachet J Bavaria J Current status and recommendations for use of the frozen elephant trunk technique: a position paper by the vascular domain of EACTS Eur J Cardiothorac Surg 2015 47 05 759 769 25769463 10.1093/ejcts/ezv085

[19] Shrestha M Haverich A Martens A Total aortic arch replacement with the frozen elephant trunk procedure in acute DeBakey type I aortic dissections Eur J Cardiothorac Surg 2017 51 01 i29 i34 28108566 10.1093/ejcts/ezw341

[20] Bashir M Shaw M Field M Repair of type A dissection—benefits of dissection rota Ann Cardiothorac Surg 2016 5 03 209 215 27386408 10.21037/acs.2016.05.09 PMC4893525

